# Supplementary material for: FERN – a Java framework for stochastic simulation and evaluation of reaction networks
Source: BMC Bioinformatics. 2008 Aug 29;9:356. doi: 10.1186/1471-2105-9-356 (PMC2553347; doi:10.1186/1471-2105-9-356)
Supplement: Additional file 1 — FERN distribution, Version 1.3. This archive contains the FERN source code and binaries as well as documentation and example models in FernML and SBML. [file 1471-2105-9-356-S1.zip › fern/doc/javadoc/fern/simulation/observer/IntervalObserver.html]

IntervalObserver


---


|  |  |  |
| --- | --- | --- |
| **Overview** \| **Package** \| **Class** \| **Use** \| **Tree** \| **Deprecated** \| **Index** \| **Help** | |  |
| **PREV CLASS**   **NEXT CLASS** | **FRAMES**    **NO FRAMES**     **All Classes** |  |
| SUMMARY: NESTED \| FIELD \| CONSTR \| METHOD | DETAIL: FIELD \| CONSTR \| METHOD |  |


---


## fern.simulation.observer Class IntervalObserver

```
java.lang.Object
  fern.simulation.observer.Observer
      fern.simulation.observer.IntervalObserver
```

**All Implemented Interfaces:**: GnuPlotObserver

**Direct Known Subclasses:**: AmountIntervalObserver, MultiAmountIntervalObserver, ReactionIntervalObserver

---

``` public abstract class IntervalObserver extends Observer implements GnuPlotObserver ```

Base class for observing certain aspects repeatedly after a given interval.
Two methods can be used to observe intervals:

- `step`: check after each step if a interval range has been passed
- `theta`: register thetas for the interval ranges

The type of the used method can be set by using `setThetaMethod(boolean)`.

Extending classes just have to implement `getEntityValue(int)` and to
pass some indices and names to to constructor.

**Author:**
:   Florian Erhard

---

**Constructor Summary**

`IntervalObserver(Simulator sim,
double interval,
String[] entityName)`
  
          Create the observer for a given simulator, a given interval, given indices with names.


| **Method Summary** | |
| --- | --- |
| `void` | `activateReaction(int mu, double tau, Simulator.FireType fireType, int times)`             Do nothing. |
| `void` | `finished()`             Adds the recorded data to the average data pool. |
| `protected abstract  double` | `getEntityValue(int i)`             Gets the actual value of the entity. |
| `double` | `getFinalValue(int entityIndex)`             Gets the last value of the given entity. |
| `double[][]` | `getRecentData()`             Gets the recorded data of the most recent simulation run. |
| `boolean` | `isPlotQuality()`             Gets whether or not to plot quality data. |
| `boolean` | `isThetaMethod()`             Using theta method means that the time intervals are registered at the simulator and the simulator invokes at this moment the method theta. |
| `void` | `setPlotQuality(boolean plotQuality)`             Sets whether or not to plot quality data. |
| `void` | `setThetaMethod(boolean thetaMethod)`             Using theta method means that the time intervals are registered at the simulator and the simulator invokes at this moment the method theta. |
| `void` | `started()`             Clears the recorded values and registers (if needed) `theta` |
| `void` | `step()`             If the `step` method is used, the data is recorded here. |
| `void` | `theta(double theta)`             If the `theta` method is used, the data is recorded here. |
| `GnuPlot` | `toGnuplot()`             Creates a new `GnuPlot` object and passes the actual observer data to it. |
| `GnuPlot` | `toGnuplot(GnuPlot gnuplot)`             Passes the actual observer data to a `GnuPlot` object. |
| `GnuPlot` | `toGnuplotRecent()`             Creates a new `GnuPlot` object and passes the recent observer data to it. |
| `GnuPlot` | `toGnuplotRecent(GnuPlot gnuplot)`             Passes the recent observer data to a `GnuPlot` object. |
| `String` | `toString()` |

**Methods inherited from class fern.simulation.observer.Observer**

`applyLabelFormat, getLabel, getLabelFormat, getNumSimulations, getPrintWriter, getSimulator, getTheta, getTitlesCommand, print, setLabelFormat, setPrintWriter, setSimulator, setTheta`

**Methods inherited from class java.lang.Object**

`clone, equals, finalize, getClass, hashCode, notify, notifyAll, wait, wait, wait`

**Methods inherited from interface fern.simulation.observer.GnuPlotObserver**

`getStyles`

**Constructor Detail**

### IntervalObserver

```
public IntervalObserver(Simulator sim,
                        double interval,
                        String[] entityName)
```

:   Create the observer for a given simulator, a given interval, given indices with names.

    **Parameters:**: `sim` - simulator: `interval` - interval: `entityName` - names of the entities


**Method Detail**

### getEntityValue

```
protected abstract double getEntityValue(int i)
```

:   Gets the actual value of the entity.

    :   **Parameters:**: `i` - index of the entity **Returns:**: value of the entity

---


### started

```
public void started()
```

:   Clears the recorded values and registers (if needed) `theta`

    :   **Specified by:**: `started` in class `Observer`

---


### activateReaction

```
public void activateReaction(int mu,
                             double tau,
                             Simulator.FireType fireType,
                             int times)
```

:   Do nothing.

    :   **Specified by:**: `activateReaction` in class `Observer`
    :   **Parameters:**: `mu` - the reaction which is supposed to fire: `tau` - the time the reaction fires (at this time `Simulator.getTime()` does not necessarily yield the firing time): `fireType` - the type of the firing: `times` - TODO

---


### finished

```
public void finished()
```

:   Adds the recorded data to the average data pool. Moreover a linear interpolation
    is done to the recorded values in order to guarantee that the time axis matches
    exactly the intervals. If the theta method has been used no interpolation is performed.

    :   **Specified by:**: `finished` in class `Observer`

---


### step

```
public void step()
```

:   If the `step` method is used, the data is recorded here.

    :   **Specified by:**: `step` in class `Observer`

---


### theta

```
public void theta(double theta)
```

:   If the `theta` method is used, the data is recorded here.

    :   **Specified by:**: `theta` in class `Observer`
    :   **Parameters:**: `theta` - moment in time

---


### isThetaMethod

```
public boolean isThetaMethod()
```

:   Using theta method means that the time intervals are registered at the simulator and
    the simulator invokes at this moment the method theta. Otherwise the algorithm steps
    are used and the values are interpolated

    :   **Returns:**: the thetaMethod

---


### setThetaMethod

```
public void setThetaMethod(boolean thetaMethod)
```

:   Using theta method means that the time intervals are registered at the simulator and
    the simulator invokes at this moment the method theta. Otherwise the algorithm steps
    are used and the values are interpolated

    :   **Parameters:**: `thetaMethod` - the thetaMethod to set

---


### toGnuplot

```
public GnuPlot toGnuplot()
                  throws IOException
```

:   **Description copied from interface: `GnuPlotObserver`**
:   Creates a new `GnuPlot` object and passes the actual observer data to it.

    :   **Specified by:**: `toGnuplot` in interface `GnuPlotObserver`
    :   **Returns:**: the created `GnuPlot` object **Throws:**: `IOException` - if gnuplot could not be accessed

---


### toGnuplot

```
public GnuPlot toGnuplot(GnuPlot gnuplot)
                  throws IOException
```

:   **Description copied from interface: `GnuPlotObserver`**
:   Passes the actual observer data to a `GnuPlot` object.

    :   **Specified by:**: `toGnuplot` in interface `GnuPlotObserver`
    :   **Parameters:**: `gnuplot` - the `GnuPlot` object to pass the data to **Returns:**: the `GnuPlot` object **Throws:**: `IOException` - if gnuplot could not be accessed

---


### getRecentData

```
public double[][] getRecentData()
```

:   Gets the recorded data of the most recent simulation run.

    :   **Returns:**: data as double array [step][entity index]

---


### toGnuplotRecent

```
public GnuPlot toGnuplotRecent()
                        throws IOException
```

:   Creates a new `GnuPlot` object and passes the recent observer data to it.
    Recent means that not the average data is used but only the recently produced.

    :   **Returns:**: the created `GnuPlot` object **Throws:**: `IOException` - if gnuplot could not be accessed

---


### toGnuplotRecent

```
public GnuPlot toGnuplotRecent(GnuPlot gnuplot)
                        throws IOException
```

:   Passes the recent observer data to a `GnuPlot` object.
    Recent means that not the average data is used but only the recently produced.

    :   **Parameters:**: `gnuplot` - the `GnuPlot` object to pass the data to **Returns:**: the `GnuPlot` object **Throws:**: `IOException` - if gnuplot could not be accessed

---


### setPlotQuality

```
public void setPlotQuality(boolean plotQuality)
```

:   Sets whether or not to plot quality data. If set to true,
    a call to `toGnuplot` adds not only the values but also one
    column in which the number of experiments (from how many values is the average of
    the regarding row calculated) is

    :   **Parameters:**: `plotQuality` - whether or not to plot qualities

---


### isPlotQuality

```
public boolean isPlotQuality()
```

:   Gets whether or not to plot quality data. If set to true,
    a call to `toGnuplot` adds not only the values but also one
    column in which the number of experiments (from how many values is the average of
    the regarding row calculated) is

    :   **Returns:**: whether or not to plot qualities

---


### getFinalValue

```
public double getFinalValue(int entityIndex)
```

:   Gets the last value of the given entity.

    :   **Parameters:**: `entityIndex` - index of the entity **Returns:**: last recorded value

---


### toString

```
public String toString()
```

:   **Overrides:**: `toString` in class `Object`


---


|  |  |  |
| --- | --- | --- |
| **Overview** \| **Package** \| **Class** \| **Use** \| **Tree** \| **Deprecated** \| **Index** \| **Help** | |  |
| **PREV CLASS**   **NEXT CLASS** | **FRAMES**    **NO FRAMES**     **All Classes** |  |
| SUMMARY: NESTED \| FIELD \| CONSTR \| METHOD | DETAIL: FIELD \| CONSTR \| METHOD |  |


---
